# Procedural Outcome and One Year Follow up of Patients Undergoing Endovascular Stenting for Coarctation of Aorta: A Single Center Study

**DOI:** 10.5681/jcvtr.2014.025

**Published:** 2014-06-30

**Authors:** Mohammad Ali Ostovan, Javad Kojuri, Maryam Mokhtaryan, Vida Razazi, Abdolali Zolghadrasli

**Affiliations:** ^1^Department of Cardiology, School of Medicine, Shiraz University of Medical Sciences, Shiraz, Iran; ^2^Students’ Research Center, Shiraz University of Medical Sciences, Shiraz, Iran; ^3^School of Management and Information, Shiraz University of Medical Sciences, Shiraz, Iran; ^4^Shiraz Cardiovascular Research Center, Shiraz University of Medical Sciences, Shiraz, Iran

**Keywords:** Coarctation of Aorta, Endovascular Stenting, Procedural Outcome

## Abstract

***Introduction:*** Coarctation of aorta is the fourth most common cardiac lesion requiring intervention. While surgery used to be the only treatment option, endovascular intervention is now considered the first option in simple coarctation lesions. Despite increased popularity, there are currently no FDA approved stents for use in coarctation of aorta and data on the outcome of this procedure is still sparse.

***Methods:*** Between October 2004 and June 2010, 33 patients who underwent treatment with Cheatham-Platinum stents for coarctation of aorta were retrospectively studied. All the patients underwent control CT scans at 6 month and echocardiography at 1 year follow-up.

***Results:*** There were 17 females and 16 males with a mean age of 26.64 ± 16.30 years (range 2-71 years). The mean stent length and balloon diameter were 3.18 ± 0.56 mm and 15.7 ± 3.12 mm respectively. We achieved an immediate success rate of 96.9% with the only complication of aortic rupture which led to our single mortality in this series. At 6 month follow up no complications were noted in the CT scans. The mean echocardiographic aortic arch gradient at one year follow up was 21.73 ± 11.06 mmHg.

***Conclusion:*** This study is one of the few cohorts of patients with stenting for coarctation of aorta in Iranian population which comprised a diverse group of patients from early childhood to elderly. It was demonstrated in this study that stenting for coarctation of aorta is a safe and effective procedure if done carefully and performed in selected patients.

## Introduction

Coarctation of Aorta constitutes 5-8% of all congenital cardiac malformations and affects males more than females.^1-3^ It is the fourth most common cardiac lesion requiring intervention either surgically or catheter-based^3^. Patients with coarctation of aorta have limited life expectancy mainly due to cardiovascular complications of sustained high blood pressure and without correction most patients die in their 5^th^ decade of life.^4,5^ Though many patients are recognized in early childhood and managed as such, 25% of patients are diagnosed in adolescence and adulthood and many more are brought to medical attention again due to re-coarctation.^1,6^ Surgery has been used since 1950s as the method of choice for patients with coarctation of aorta, but since 1980s initially balloon angioplasty came into practice and finally in 1996 the first use of stents for the management of coarctation of aorta was reported.^1^ There are currently several covered and non-covered stents used in clinical practice. Despite the presence of several studies reporting the safety and efficacy of stenting and the inclusion of catheter-based methods in American Heart Association (AHA) guidelines, there is still no concrete data regarding the outcome of this technique due to lack of clinical trials and there are concerns with regard to serious procedural and long-term complications.^1,3,7^ Currently no stents are approved by the Food and Drug Administration (FDA) and all the available stents are used off-label.^1^ In this paper, we report our single center experience of coarctation of aorta stenting in a wide range of patients using the covered and non-covered Cheatham-Platinum (CP) stents and the one year follow-up of these patients.

## Material and methods

### Patients and data collection

Between October 2004 and June 2010, 33 consecutive patients who underwent stenting for coarctation of aorta in Kowsar Heart Hospital in Shiraz, Iran were included in this study. The available medical records of the patients were studied retrospectively. The patients were all above 1 year old and had either arm to leg systolic pressure gradient above 20 mmHg or had lower gradient accompanied with systemic hypertension defined as greater than 95^th^ percentile for age, height and gender.^3,8,9^ All the patients underwent magnetic resonance imaging (MRI) or computed tomographic (CT) imaging and proceeded to stenting if their anatomies were deemed suitable by their caring interventional cardiologists. Informed consents were taken from the patients or their caregivers before the procedure.

### Stenting technique

**Figure 1:**
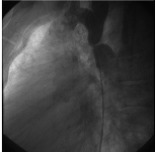
Angiogram in lateral view of a 23 year old male before stenting.

**Figure 2:**
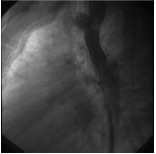
The same patient in figure 1 after stenting

All patients underwent cardiac catheterization and biplane aortography under conscious sedation, and in the case of children and adolescents below the age 18 under deep sedation in the care of an anesthesiologist. The stenting technique is explained in detail elsewhere but we present in brief the procedure.^3,6,7,10-12^ After delineation of the coarctation anatomy and measurement of the coarctation segment in length from distal of the origin of the last arch vessel to the immediate non-coarcted segment in descending aorta, the diameter of the stent was determined according to the diameter of the aorta at the diaphragm. Primary stenting with CP stent (NuMED CP stent, Heart Medical Europe BV, Best, the Netherlands) was done in all cases. Covered stents were chosen, at operator’s discretion, if the patient’s age was above 40, near-atresia coarctation or complex anatomy was detected with the possibility of aortic wall injury, otherwise a bare metal stent was deployed.^12,13^ The stent was hand crimped on a balloon and then it was delivered using the balloon in balloon (BiB) catheter (NuMED Inc., Heart Medical Europe BV, Best, the Netherlands). Post-stenting dilation was used only in case of stent under-deployment. After stent delivery, a biplane aortogram was performed to check for any evidence of aortic wall injury (Figure 1  and 2 ). The procedure was defined as successful if the gradient across the coarcted segment fell to less than 10 mmHg with an increase in the luminal diameter of the coarcted segment to more than 90% of the adjacent aorta without any immediate aortic wall injury.^3,14^ Finally hemostasis at femoral insertion site was achieved via manual compression.

### Follow-up

The patients were observed overnight, their blood pressure and femoral pulses were monitored frequently and they were discharged if there were no complications. The patients were all visited in 1, 3, 6 and 12 months interval and yearly thereafter. The patients underwent a control CT scan after 6 months and an echocardiography to determine the peak systolic gradients across the aortic arch at each visit using Doppler ultrasound. Although CP stents are MRI compatible, we decided to use CT scan to minimize artifacts, allowing us to better apprehend aortic wall complications. Aspirin was given to patients for at least 6 months.

### Statistical Analysis

The results are expressed as the mean ± SD for continuous variables and as counts or percentages for nominal variables. Student’s t test was used for continuous variables and Pearson’s correlation coefficient is used for correlation studies. All data were analyzed using SPSS version 18 (Chicago, IL, USA). P value < 0.05 was considered significant.

## Results

Patient population: A total of 33 patients with the mean age of 26.64 ± 16.30 years (range 2-71 years) were included in this study. There were 17 females and 16 males. The mean age of females was 22 ± 3.19 years (range 2-58 years) and the mean age of males was 27.44 ± 4.77 years (range 3-71 years) which was not significantly different (p= 0.34).

Angiographic data: The mean stent length and balloon diameter were 3.18 ± 0.56 cm and 15.7 ± 3.12 mm respectively (range 2.2-4.5 cm and 8-24 mm respectively). The mean stent length was 3.21 ± 0.48 cm for females and 3.159 ± 0.63 cm for males which was not different statistically (p= 0.78). The mean balloon diameter was 16.75 ± 2.81 mm for females and 14.71 ± 3.15 mm for males which again was not significantly different (p= 0.06), though it showed a trend toward smaller size for male patients. In 26 patients (78.78%) bare metal stents were used and in 7 patients (21.22%) covered stents were used; 2 patients were older than 40 years, 4 patients had near-atretic anatomy and 1 patient had aneurysmal dilation of adjacent aorta.

Procedural success and complications: After stent deployment, trans-aortic gradient was measured by catheter pullback and a control angiogram was performed. In all the procedures the gradient fell below 10 mmHg and the stented segment diameter increased more than 90% compared to adjacent aorta. The mean post-stenting gradient was 2.5 ± 3.92 mmHg. No stent migration, stent under-deployment, dissection or pseudoaneurysm was noted. In one 26 year old female patient, aortic rupture occurred. Although she had a tight coarctation segment, because of lack of associated bicuspid aortic wall or aortopathy and simple anatomy, bare metal stent was chosen. The adjacent aorta was measured 16 mm at biplane angiography and after dilation of the bare metal stent with an 18mm balloon, contrast extravasation was noted. Due to lack of back-up covered stent in the angiography unit, an attempt was made to stabilize the patient with balloon inflation and transfer of the patient to operation room emergently, but unfortunately the patient died in the operation room.

### Follow-up

The patients were discharged without any complications the day after the procedure (except the one mortality). At 6-month follow up no complications were noted in the CT scan obtained for each patient. The mean echocardiographic aortic arch gradient at one year follow up was 21.73 ± 11.06 mmHg (range 8-60 mmHg). The mean gradient was 21.13 ± 12.47 mmHg for males and 22.29 ± 9.91 mmHg for females which was not statistically different (p value = 0.76). In this echocardiography study, 6 patients (18.2%) had gradients below 10 mmHg, 13 patients (39.4%) had gradients between 10 and 20 mmHg, another 6 patients (18.2%) had gradients between 20 and 30 mmHg and 8 patients (24.2%) had gradients above 30 mmHg. The latter group of patients subsequently underwent re-dilation with a high pressure balloon if they were hypertensive.

A correlation study found no relation between age, stent length and balloon diameter with follow-up echocardiographic gradients (Table 1).

**Table 1:** Correlation study of stent length, balloon diameter and age with 1 year follow-up gradient

	**Correlation coefficient**	**P value**
Stent length	0.189	0.44
Balloon diameter	0.155	0.18
Age	0.187	0.08

## Discussion

Reports regarding the safety and outcome of coarctation of aorta stenting are still being published in the literature. This is the second largest report of procedural outcome and 1 year follow-up of patients undergoing stenting for coarctation of aorta in Iran and is unique to include a wide range of patients from early childhood through late elderly.^15,16^ This report shows the overall efficacy and safety of this procedure in a diverse group of patients. We achieved an immediate success rate of 96.9% with the only complication of aortic rupture which led to our single mortality in this series. No complications were noted in the 6 month follow-up CT scan and in the 1 year follow-up 75.8% of patients had gradients below 30 mmHg.

Coarctation of aorta, which accounts for 0.04% of all live births, has wide morphologic presentations ranging from a simple tubular narrowing to complete arch interruption.^2,3^ While surgery used to be the only treatment option, endovascular stenting usage has gained popularity and with increased operator experience and higher technologies, this has translated into higher success with lower complication rates.^9,11^ Although some small cohort regarding the profile and outcome of this relatively new procedure has been published, data from large multicenter studies are still scarce. In one landmark study, the Congenital Cardiovascular International Study Consortium (CCISC) published the retrospective survey of 627 stent implants in 565 procedures across 14 centers from 1989 to 2002. In this study a 98% immediate success rate was reported with a 14.3% complication rate including 2 deaths. Other complications noted were aneurysm formation (2.25%), dissection (1.5%), stent migration (4.8%), arterial access injury (2.6%) and stroke (1%).^1,8^ In an ongoing prospective trial (COAST), stenting was successfully done in 104 out of 105 patients with stent migration occurring in the remaining case. The overall complication was 34%, much higher than CCISC study, but no serious complications or deaths were noted, implicating increased experience in this field.^6^

One drawback of stenting is the possibility of complications especially of serious ones. The major complications of stenting are aortic wall injury in the form of aneurysms and dissections.^17^ They are mostly reported in patients with severe aortopathy like those above 65 years and those with associated biscuspid aortic valve.^17^ The complication rate with stenting seems to be 1-5%, well below 2-20% observed with balloon angioplasty alone.^3^ The major morbidity and complication rate in patients undergoing surgery is estimated to be 9%.^1^

In our study we had a high immediate success rate and faced only one immediate complication which is quite comparable to other studies which almost show immediate success rate of 100% but serious complications ranging from 0-11%.^6,8,10,12,13,18-20^ Aortic rupture is the most dreaded complication of coarctation stenting and is reported in other studies as well.^8,10,12,13^ In CCIS two patients developed aortic rupture and underwent emergency surgery.^8^ In a cohort of 22 patients, 1 patient developed aortic rupture during stenting with covered stent for whom another covered stent was deployed successfully.^13^ In another cohort of 45 patients, one of them developed aortic rupture which was successfully managed with covered stent.^10^ This complication is assumed to occur mostly in the elderly while our patient was 26 years old.^13^ Risk factors for this complication include performing pre-stent dilation; location of the coarctation in abdominal aorta and age above 40 years.^12^ The main cause of death in our patient was the absence of back-up covered stents in the catheterization unit. This should prompt interventionists to ask for the availability of back-up covered stents in case this serious complication occur.^3^ While some authors advocate the prophylactic use of covered stents to avoid this complication, others have found no evidence to support this notion.^3,10,12,13^ The rather low rate of complications in our study can be ascribed to careful patient selection, avoidance of pre-stenting dilation and meticulous use of balloons for post-stenting dilation of the coarcted segment. The operator should be very cautious in the passage of wires and use of balloons when dealing with coarctation of aorta to prevent aortic wall injury.

There are currently no FDA approved stents for aortic coarctation^1^. An ideal coarctation stent should expand from 12 to 24mm in diameter with minimal foreshortening or loss of radial strength at maximal diameter.^1^ CP stents with 8 zigs conformation, have low profile with a maximum diameter of 22mm and foreshortening no more than 20% at maximum diameter and are designed to minimize aortic wall injury which make them suitable stents for implantation in coarcted segments and thus were used in all our procedures.^1,11,12,16,21^ Bare metal stents have the advantages of greater ability for later redilation, deployment through smaller delivery sheaths and lower possibility of obstruction of aortic side branches, while covered stents have the advantages of lower aortic wall complications and are used to exclude the aneurysmal portion of the aorta from circulation.^11,13,17^

In 1 year follow-up we chose echocardiographic arch gradients as efficacy outcome. There are currently many variables used to determine residual coarctation. Directly measured pressures in catheterization units are too invasive and inconsistent based on anesthesia method^1^. There is also poor correlation between resting or exercise induced systemic hypertension and arch obstruction.^1^ Possibly the most accurate determinant of residual coarctation or recoarctation is upper body to lower body systolic pressure gradient^1^. Considering the retrospective nature of this study and the difficulties in routine measurements of upper and lower body blood pressures, we opt to use the echocardiographic data instead which has been shown to provide a reliable index of re-coarctation.^22^ At 1 year follow-up 75.8% of our patients had gradients below 30 mmHg which is an acceptable gradient^11^. Few studies have used echocardiographic measurements for follow-up gradients. In one study the mean gradient was 23.4 ± 4.3 mmHg at an average follow-up of 52.6 months which is comparable to our series of 21.73 ± 11.06 mmHg.^11^ In another cohort the gradient at 6 month follow-up was 32 ± 19 mmHg.^10^ In another study invasive measurement was done which showed 3 out of 22 patients had re-coarctation and needed re-intervention.^13^ In a small cohort of 16 patients, one of them needed re-intervention after 14 months due to re-coarctation.^4^ In some other smaller cohorts using invasive hemodynamic studies, no need for intervention was seen at 1-2 years follow-up.^19,23^

In our follow-up study no patients demonstrated late complications, while in a cohort of 235 patients the late aortic wall complication rate was 16%^3^. Late aortic wall aneurysms seem to occur more frequently in native coarctation than re-coarctation.^3^

There are currently no randomized trials directly comparing surgical methods with endovascular methods, but an observational study by CCISC concluded that stent patients had significantly lower complications compared to surgery and balloon angioplasty patients, while at short-term and intermediate follow-up surgical and stenting patients achieved the same hemodynamic outcomes which were both superior to balloon angioplasty patients.^9^ In another prospective observational study with 11 cases, both surgery and stenting achieved the same hemodynamic outcomes, with stenting patients having shorter hospital stay and surgery patients requiring fewer antihypertensive therapies.^11^ Whereas there is few debate that stenting should be the first choice in patients above 10 years old or when there is ventricular dysfunction and/or other comorbidities, surgery is preferred in complex anatomies with additional cardiac anomalies, large aneurysms or access site problems.^3,5,10^ Currently the most common criteria for stenting have been previous surgical repair, high surgical risk and refusal of consent to surgery.^20^

Our study has many limitations with the main attributive factor being the retrospective nature of the study. It is not randomized, there is no comparison group and is performed on a relatively small number of highly selected patients without complex anatomy and so no comparison can be made with surgical series.

This study is one of the few reports of patients with stenting for coarctation of aorta in an Iranian population which comprised of a diverse group of patients from early childhood to elderly. Although we faced one mortality in this study which is rarely reported in other series, but no other immediate or late complications were noted. As a result, it was demonstrated that stenting is a safe and effective procedure if done carefully and performed in selected patients in well-equipped centers.

## Acknowledgements

This project is supported by deputy dean of School of Medicine based on research project number 2679 and sponsored by deputy chancellor of Shiraz University of Medical Sciences.

## Ethical issues

This study was approved by our local Ethics Committee.

## Competing interests

Authors declare no conflict of interest in this study.

## References

[1] Ringel RE, Gauvreau K, Moses H, Jenkins KJ (2012). Coarctation of the Aorta Stent Trial (COAST): study design and rationale. Am Heart J.

[2] Kenny D, Hijazi ZM (2011). Coarctation of the aorta: from fetal life to adulthood. Cardiol J.

[3] Gewillig M, Budts W, Boshoff D, Maleux G (2012). Percutaneous interventions of the aorta. Future Cardiol.

[4] Wheatley  GH 3rd, Koullias GJ, Rodriguez-Lopez JA, Ramaiah VG, Diethrich EB (2010). Is endovascular repair the new gold standard for primary adult coarctation?. Eur J Cardiothorac Surg.

[5] Jurcut R, Daraban AM, Lorber A, Deleanu D, Amzulescu MS, Zara C (2011). Coarctation of the aorta in adults: what is the best treatment? Case report and literature review. J Med Life.

[6] Ringel RE, Vincent J, Jenkins KJ, Gauvreau K, Moses H, Lofgren K (2013 Oct 1). Acute outcome of stent therapy for coarctation of the aorta: results of the coarctation of the aorta stent trial. Catheter Cardiovasc Interv.

[7] Moltzer E, Roos-Hesselink JW, Yap SC, Cuypers JA, Bogers AJ, de Jaegere PP (2010). Endovascular stenting for aortic (re)coarctation in adults. Neth Heart J.

[8] Bozzani A, Odero A (2012). Stent implantation in the native and recurrent aortic coarctation in children. Am J Cardiol.

[9] Forbes TJ, Kim DW, Du W, Turner DR, Holzer R, Amin Z (2011). Comparison of surgical, stent, and balloon angioplasty treatment of native coarctation of the aorta: an observational study by the CCISC (Congenital Cardiovascular Interventional Study Consortium). J Am Coll Cardiol.

[10] Erdem A, Akdeniz C, Sarıtaş T, Erol N, Demir F, Karaci AR (2011). Cheatham-Platinum stent for native and recurrent aortic coarctation in children and adults: immediate and early follow-up results. Anadolu Kardiyol Derg.

[11] San Norberto García EM, González-Fajardo JA, Gutiérrez V, Fernández B, San Román A, Vaquero C (2010). Open surgical repair and endovascular treatment in adult coarctation of the aorta. Ann Vasc Surg.

[12] Godart F (2011). Intravascular stenting for the treatment of coarctation of the aorta in adolescent and adult patients. Arch Cardiovasc Dis.

[13] Tanous D, Collins N, Dehghani P, Benson LN, Horlick EM (2010). Covered stents in the management of coarctation of the aorta in the adult: initial results and 1-year angiographic and hemodynamic follow-up. Int J Cardiol.

[14] Bentham JR, English K, Ballard G, Thomson JD (2013). Effect of interventional stent treatment of native and recurrent coarctation of aorta on blood pressure. Am J Cardiol.

[15] Molaei A, Merajie M, Mortezaeian H, Malakan Rad E, Haji Heidar Shemirani R (2011). Complications of Aortic Stenting in Patients below 20 Years Old: Immediate and Intermediate Follow-Up. J Tehran Heart Cent.

[16] Sohrabi B, Jamshidi P, Yaghoubi A, Habibzadeh A, Hashemi-Aghdam Y, Moin A (2014). Comparison between covered and bare Cheatham-Platinum stents for endovascular treatment of patients with native post-ductal aortic coarctation: immediate and intermediate-term results. JACC Cardiovasc Interv.

[17] Thanopoulos BD, Giannakoulas G, Giannopoulos A, Galdo F, Tsaoussis GS (2012 15). Initial and six-year results of stent implantation for aortic coarctation in children. Am J Cardiol.

[18] Shennib H, Rodriguez-Lopez J, Ramaiah V, Wheatley G, Kpodonu J, Williams J (2010). Endovascular management of adult coarctation and its complications: intermediate results in a cohort of 22 patients. Eur J Cardiothorac Surg.

[19] Thanopoulos BD, Hadjinikolaou L, Konstadopoulou GN, Tsaousis GS, Triposkiadis F, Spirou P (2000). Stent treatment for coarctation of the aorta: intermediate term follow up and technical considerations. Heart.

[20] Baykan A, Karagöz T, Celiker A (2009). Endovascular stent implantation for coarctation of the aorta in children and young adults: intermediate follow-up results from Turkey. Turk J Pediatr.

[21] Ewert P, Schubert S, Peters B, Abdul-Khaliq H, Nagdyman N, Lange PE (2005). The CP stent--short, long, covered--for the treatment of aortic coarctation, stenosis of pulmonary arteries and caval veins, and Fontan anastomosis in children and adults: an evaluation of 60 stents in 53 patients. Heart.

[22] Hajsadeghi S, Fereshtehnejad SM, Ojaghi M, Bassiri HA, Keramati MR, Chitsazan M (2012). Doppler echocardiographic indices in aortic coarctation: a comparison of profiles before and after stenting. Cardiovasc J Afr.

[23] Harrison DA, McLaughlin PR, Lazzam C, Connelly M, Benson LN (2001). Endovascular stents in the management of coarctation of the aorta in the adolescent and adult: one year follow up. Heart.

